# Supplementary material for: A Comparative Study of Immunogenicity, Antibody Persistence, and Safety of Three Different COVID-19 Boosters between Individuals with Comorbidities and the Normal Population
Source: Vaccines (Basel). 2023 Aug 17;11(8):1376. doi: 10.3390/vaccines11081376 (PMC10459403; doi:10.3390/vaccines11081376)
Supplement: Supplementary file 1 [file vaccines-11-01376-s001.zip › vaccines-2495850-supplementary.pdf]

Supplementary Table S1. Median and IQR of anti-SARS-CoV-2 Spike, anti-SARS-CoV-2 RBD, and neutralizing antibody fold rise on days 28, 60, 90, and 180 in healthy people.

	Day 28		Day 60		Day 90		Day 180	
	**Median (IQR)**	**P**	**Median (IQR)**	**P**	**Median (IQR)**	**P**	**Median (IQR)**	**P**
**Anti-SARS-CoV-2 Spike antibody median fold rise (IQR)**								
**Age**								
≤ 40	1.84 (1.62)	0.28	1.03 (3.12)	0.17	0.80 (2.06)	0.12	0.64 (2.41)	**0.04**
> 40	4.08 (24.58)		4.75 (16.27)		1.39 (9.66)		3.14 (10.32)	
**Gender**								
Female	2.19 (3.62)	0.9	1.58 (6.88)	0.99	0.88 (2.44)	0.69	1.22 (3.95)	0.77
Male	3.07 (26.53)		2.11 (26.61)		1.27 (12.17)		2.11 (15.05)	
**COVID-19 History**								
No	2.04 (12.24)	0.2	1.75 (10.06)	0.4	1.24 (7.58)	0.37	1.24 (8.90)	0.98
Yes	3.12 (70.67)		7.70 (14.05)		0.81 (2.23)		2.14 (2.93)	
**Booster**								
BP	2.55 (20.21)		3.34 (26.99)		3.50 (8.80)		4.08 (8.42)	
BPa	2.71 (31.53)	0.39	1.53 (11.34)	0.17	0.74 (1.61)	0.11	1.15 (2.43)	0.26
BB	2.28 (4.78)		2.11 (7.26)		1.22 (5.35)		2.76 (8.69)	
**Anti-SARS-CoV-2 Neutralizing antibody median fold rise (IQR)**								
**Age**								
≤ 40	1.01 (0.05)	0.09	1.05 (0.06)	0.10	1.02 (0.20)	0.18	1.01 (0.16)	0.11
> 40	1.15 (1.22)		1.2 (1.07)		1.19 (1.51)		1.43 (2.05)	
**Gender**								
Female	1.04 (0.13)	0.39	1.05 (0.16)	0.76	1.04 (0.27)	0.57	1.04 (0. 22)	0.68
Male	1.77 (2.00)		1.39 (2.00)		1.13 (2.07)		1.8 (2.20)	
**COVID-19 History**								
No	1.08 (1.01)	0.07	1.09 (0.86)	0.06	1.07 (1.09)	0.48	1.05 (1.07)	0.90
Yes	1.01 (0.74)		1.05 (0.96)		1.03 (1.55)		1.01 (1.89)	
**Booster**								
BP	1.49 (5.38)		1.51 (5.11)		1.56 (5.29)		1.44 (5.81)	
BPa	1.05 (0.51)	0.06	1.06 (0.71)	**0.04**	1.00 (0.82)	0.11	1.02 (0.71)	0.32
BB	1.04 (0.46)		1.02 (0.40)		1.12 (0.84)		1.14 (1.48)	
**Anti-SARS-CoV-2 RBD antibody median fold rise (IQR)**								
**Age**								
≤ 40	1.84 (2.62)	0.52	0. 51(4.95)	0.19	0.40 (1.90)	0.17	0.46 (4.07)	0.11
> 40	2.69 (13.33)		2.69 (13.33)		1.56 (8.47)		1.09 (6.41)	
**Gender**								
Female	1.88 (5.10)	0.16	2.12 (5.48)	0.66	0.85 (45.30)	0.88	1.04 (0.22)	0.89
Male	3.46 (136.07)		1.35 (22.53)		0.80 (13.79)		1.84 (2.20)	
**COVID-19 History**								
No	2.82 (20.89)	**0.02**	2.12 (8.96)	**0.01**	1.56 (8.69)	**0.02**	0. 89 (5.14)	0.13
Yes	1.17 (0.91)		0.47 (2.49)		0.19 (0.44)		0.31 (0.81)	
**Booster**								
BP	6.81 (155.93)	**0.02**	5.64 (25.34)	0.07	5.20 (8.31)	0.10	2.17 (6.09)	0.16

BPa	2.02 (11.16)	1.66 (5.60)	0.85 (3.67)	0.52 (1.09)
BB	1.49 (4.67)	0.61 (5.51)	0.51 (4.43)	0.80 (24.65)

HC: healthy control. BP: BBIBP-CorV /PastoCovac-Plus, BPa: BBIBP-CorV /PastoCovac, BB: BBIBP-CorV /BBIBP-CorV . Bold P values are shown as significant.
